# Modulation of the Proliferative Pathway, Neuroinflammation and Pain in Endometriosis

**DOI:** 10.3390/ijms241411741

**Published:** 2023-07-21

**Authors:** Livia Interdonato, Ylenia Marino, Ramona D’Amico, Marika Cordaro, Rosalba Siracusa, Daniela Impellizzeri, Francesco Macrì, Roberta Fusco, Salvatore Cuzzocrea, Rosanna Di Paola

**Affiliations:** 1 Department of Chemical, Biological, Pharmaceutical and Environmental Sciences, University of Messina, 98166 Messina, Italy; interdonatol@unime.it (L.I.);; 2 Department of Biomedical, Dental and Morphological and Functional Imaging, University of Messina, Consolare Valeria, 98100 Messina, Italy; 3 Department of Veterinary Sciences, University of Messina, Viale Anunziata, 98168 Messina, Italy

**Keywords:** molecular mechanism, oxidative stress, natural compound

## Abstract

Endometriosis is a chronic disease characterized by pelvic inflammation. This study aimed at investigating the molecular mechanisms underlying the pathology and how they can be modulated by the administration of a natural compound, *Actaea racemosa* (AR). We employed an in vivo model of endometriosis in which rats were intraperitoneally injected with uterine fragments from donor animals. During the experiment, rats were monitored by abdominal high-frequency ultrasound analysis. AR was able to reduce the lesion’s size and histological morphology. From a molecular point of view, AR reduced hyperproliferation, as shown by Ki-67 and PCNA expression and MAPK phosphorylation. The impaired apoptosis pathway was also restored, as shown by the TUNEL assay and RT-PCR for Bax, Bcl-2, and Caspase levels. AR also has important antioxidant (reduced Nox expression, restored SOD activity and GSH levels, and reduced MPO activity and MDA levels) and anti-inflammatory (reduced cytokine levels) properties. Moreover, AR demonstrated its ability to reduce the pain-like behaviors associated with the pathology, the neuro-sensitizing mediators (c-FOS and NGF) expression, and the related central astrogliosis (GFAP expression in the spinal cord, brain cortex, and hippocampus). Overall, our data showed that AR was able to manage several pathways involved in endometriosis suppression.

## 1. Introduction

Endometrium-like stromal and glandular cells proliferate outside of the uterus in the persistent, excruciatingly painful illness known as endometriosis [1]. It is an inflammatory, estrogen-dependent condition that affects up to 40% of women undergoing fertility treatments and 7–11% of women during their reproductive years [2]. Patients’ symptoms include severe dysmenorrhea, ongoing pelvic pain, and infertility [3]. Although several ideas have been published on the etiology of endometriosis, its pathophysiology is still unknown. The retrograde menstruation theory put forward by Sampson is the most frequently accepted [4]. This idea states that endometrial gland fragments are the source of endometriosis. During menstruation, retrograde migration allows unattached endometrial glands, cells, and debris to enter the peritoneum, where they can develop and implant. Hyperproliferation, poor apoptosis, and an inflammatory microenvironment all contribute to the formation of endometriotic lesions [5,6]. Therefore, cell death is crucial for maintaining homeostasis and eliminating surplus or unhealthy cells. A crucial defense mechanism against the development of endometriosis involves maintaining differentiated tissue through a delicate balance between cell proliferation and apoptosis. Recent research highlights the significance of the oxidative imbalance, inflammatory reactions, and associated persistent pain condition both at the lesion site and in the peritoneum [7,8]. Chronic pelvic pain is caused by an intensified inflammatory response in the peritoneum, which activates sensory nerves [9]. The central sensitization process is triggered by the stimulation of peripheral nerve terminals, which transmit the impulses to the spinal cord and brain [10,11]. Black cohosh (Cimicifuga racemose or Actaea racemosa (AR)) is a widely employed herbal treatment in Asia, Europe, America, and Australia for many women’s health disorders [12,13,14,15]. Additionally, it is usually employed as a food supplement [16]. Historically, it was assumed to relieve menstruation pain by Native Americans [17]. Chemically, it has been characterized by its components [18,19,20]. These studies showed that the main constituents of AR are phenols, glycosides, alkaloids, and flavonoids, among others. In minor quantities, also present are aromatic acids (ferulic and iso ferulic acids, and methyl esters of caffeic acid), resin, cinnamic acid esters (cimicifugic acid A–F, cimicifugic acid, cimiracemates A–D, piscidic acid, fukiic acid, and fukinolic acid), fatty acids, starch, phytosterol, and sugar [20]. The biological activities shown by AR have been ascribed to alkaloids (anagyrine, quinolinic and quinolizidine alkaloids, baptifolin, methylcystine, magnoflorine, and methyl serotonin), saponins, and triterpenes (23-epi-26- deoxyactein and actein) [21,22,23]. In particular, AR works on the cell cycle by inducing the G_0_/G_1_ phase arrest at low concentration and the G_2_/M arrest and apoptosis at high concentration in vitro in hepatocellular carcinoma. In vivo tests showed that AR administration reduced the growth of implanted mouse tumors [24]. Moreover, it has important antioxidant power, acting as a radical scavenger and restoring the activities of antioxidant phase II enzymes [25]. Additionally, it has been demonstrated that AR acts on the central nervous system (CNS) by reducing neuroinflammation [26]. Recent studies exposed the beneficial effect of AR for treating postmenopausal symptoms [27]. Although previous research investigated the effects of AR on various gynecological conditions such as hyperandrogenism, polycystic ovary syndrome, and oligo/amenorrhea, the limited quantity of data warranted further research [28]. Based on the literature data about the biological effect of AR, this paper aimed to test whether AR administration would modulate the molecular mechanism of endometriosis in both lesions growth and CNS inflammation.

## 2. Results

### 2.1. Effect of AR Administration on Endometriotic Lesions Growth

**Figure 1:**
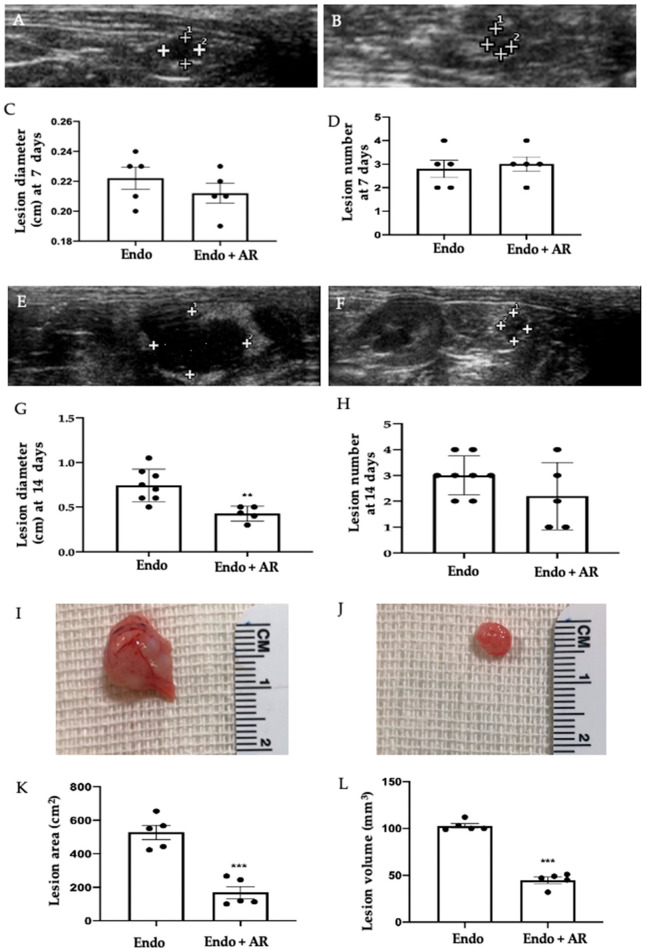
AR administration reduced the growth of endometriotic lesions: Abdominal high-frequency ultrasound (hUS) analysis seven days after the endometriosis induction: Endo (**A**), Endo + AR (**B**), lesion diameter (**C**), lesion number (**D**); hUS analysis fourteen days after the endometriosis induction: Endo (**E**), Endo + AR (**F**), lesion diameter (**G**), lesion number (**H**); macroscopic analysis: Endo (**I**), Endo + AR (**J**), lesion area (**K**), lesion volume (**L**). The Kolmogorov–Smirnov test was applied to verify the normal distribution of the data, and then the *t*-test was applied. A *p*-value of less than 0.05 was considered significant. ** *p* < 0.01 vs. Endo; *** *p* < 0.001 vs. Endo.

Abdominal high-frequency ultrasound (hUS) analysis was employed to monitor the disease. Seven days after the endometriotis induction, no differences were detected between the groups (Figure 1A, Endo group; Figure 1B, Endo + AR group) in lesion numbers (Figure 1D, Endo group (2.8 ± 0.37), Endo + AR group (3 ± 0.32), *p* = 0.6938) and diameter (Figure 1C, Endo group (0.22 ± 0.01), Endo + AR group (0.21 ± 0.01), *p* = 0.342). Seven days later, the analysis was repeated before the sacrifice. At this timepoint, the hUS exam showed an increased lesion diameter in the Endo group (Figure 1E,G (0.74 ± 0.08), *p* = 0.0015) as compared to the animals treated with AR (Figure 1F,G (0.43 ± 0.104), *p* = 0.0015). No difference between the groups was found in lesion number at this timepoint either (Figure 1H, Endo group (3 ± 0.34), Endo + AR group (2.2 ± 0.58), *p* = 0.2610). Once the animals were sacrificed, a macroscopical analysis was conducted. Lesions harvested from the Endo group (Figure 1I) showed higher area (Figure 1K, 527.4 ± 42.50, *p* = 0.0002) and volume (Figure 1L, 102.8 ± 2.4, *p* < 0.0001) as compared to the samples from the AR-administered rats (Figure 1J,K, 168.8 ± 35.9, *p* = 0.0002; and Figure 1L, 44.8 ± 3.35, *p* < 0.0001).

### 2.2. Effect of AR Administration on Lesion Morphology and Cellular Proliferation

**Figure 2:**
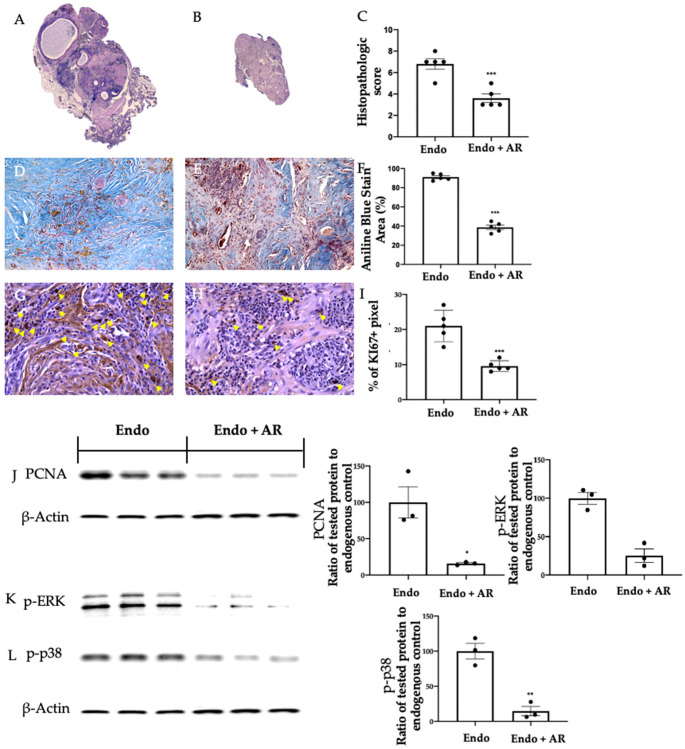
AR administration on lesion morphology, fibrosis, and cellular hyperproliferation: Histological analysis: Endo (**A**), Endo + AR (**B**), histopathologic score (**C**); Masson’s trichrome staining (magnification 20×): Endo (**D**), Endo + AR (**E**), aniline blue stain area (**F**); immunohistochemical analysis of Ki-67 expression (magnification 40×): Endo (**G**), Endo + AR (**H**), graphical quantification of Ki-67 expression (**I**); Western blot analysis of PCNA (**J**), p-ERK (**K**), p-p38 (**L**). The Kolmogorov–Smirnov test was applied to verify the normal distribution of the data, and then the *t*-test was applied. A *p*-value of less than 0.05 was considered significant. * *p* < 0.05 vs. Endo; ** *p* < 0.01 vs. Endo; *** *p* < 0.001 vs. Endo.

Hematoxylin and eosin staining of the samples showed epithelial glands and stroma in the once from the Endo group (Figure 2A, (6.8 ± 0.49)), while AR administration reduced the histopathological score (Figure 2B,C (3.6 ± 0.4), *p* = 0.001). Masson’s trichrome staining was done to evaluate tissue fibrosis. Collagen deposition was significantly reduced in the Endo + AR group (Figure 2E,F (38.6 ± 2.34), *p* < 0.0001), as compared to the Endo rats (Figure 2D,F (91 ± 3.39), *p* < 0.0001). Cellular proliferation was evaluated by immunohistochemical and Western blot analysis. Ki-67 was found to be significantly reduced by AR administration (Figure 2H,I (9.6 ± 0.68), *p* = 0.0006), as compared to the Endo group (Figure 2G,I (21 ± 2), *p* = 0.0006). The same trend was followed by PCNA level, which was higher in the Endo group and reduced by AR administration (Figure 2J, Endo group (100 ± 21.38), Endo + AR group (15.82 ± 1.18), *p* = 0.0171). Western blot analysis was also employed to evaluate the MAPKs. We found reduced ERK (Figure 2K, Endo group (100 ± 7.86), Endo + AR group (25.26 ± 8.69), *p* = 0.0031) and p38 (Figure 2L, Endo group (100 ± 11.22), Endo + AR group (14.66 ± 6.64), *p* = 0.0028) phosphorylation in samples from the Endo + AR group as compared to the Endo group. 

### 2.3. Effect of AR Administration on Apoptosis

**Figure 3:**
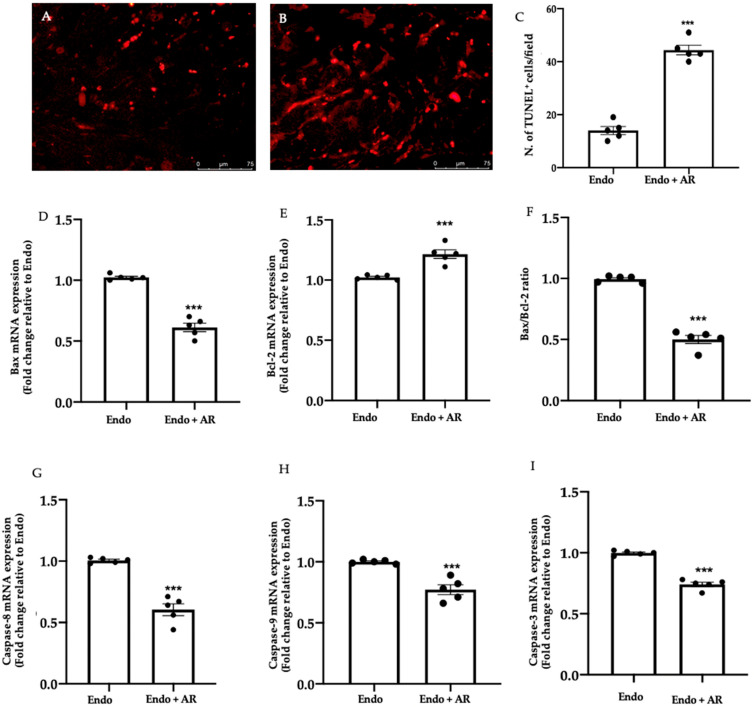
AR administration restored the apoptosis pathway: TUNEL assay: Endo (**A**), Endo + AR (**B**), number of TUNEL^+^ cells (**C**); RT-PCR analysis: Bax (**D**), Bcl-2 (**E**) mRNA levels, Bax/Bcl-2 ratio (**F**), Caspase-8 (**G**), Caspase-9 (**H**), and Caspase-3 (**I**) mRNA levels. The Kolmogorov–Smirnov test was applied to verify the normal distribution of the data, and then the *t*-test was applied. A *p*-value of less than 0.05 was considered significant. *** *p* < 0.001 vs. Endo.

TUNEL assay showed an increased number of apoptotic cells in samples from the Endo + AR group (Figure 3B,C (44.4 ± 1.83), *p* < 0.0001), as compared to the Endo group (Figure 3A,C (14 ± 1.52), *p* < 0.0001). Apoptosis was also investigated by RT-PCR. We found a reduced expression of Bax mRNA level in the Endo + AR samples (1.02 ± 0.01, *p* < 0.0001), as compared to the Endo one (Figure 3D (0.61 ± 0.04), *p* < 0.0001), while Bcl-2 had the opposite trend (Figure 3E, Endo group (1.02 ± 0.01), Endo + AR group (1.22 ± 0.04), *p* = 0.0007); thus, AR administration reduced the Bax/Bcl-2 ratio (Figure 3F, Endo group (1 ± 0.01), Endo + AR group (0.5 ± 0.03), *p* < 0.0001). Additionally, we investigated Caspase mRNA expression. In particular, the Endo + AR group showed reduced Caspase-8 (Figure 3G, Endo group (1.01 ± 0.01), Endo + AR group (0.6 ± 0.05), *p* < 0.0001), Caspase-9 (Figure 3H, Endo group (1 ± 0.01), Endo + AR group (0.77 ± 0.04), *p* = 0.0005), and Caspase-3 (Figure 3I, Endo group (1 ± 0.01), Endo + AR group (0.74 ± 0.02), *p* < 0.0001), as compared to the Endo group.

### 2.4. Effect of AR Administration on Oxidative Stress and Inflammation

**Figure 4:**
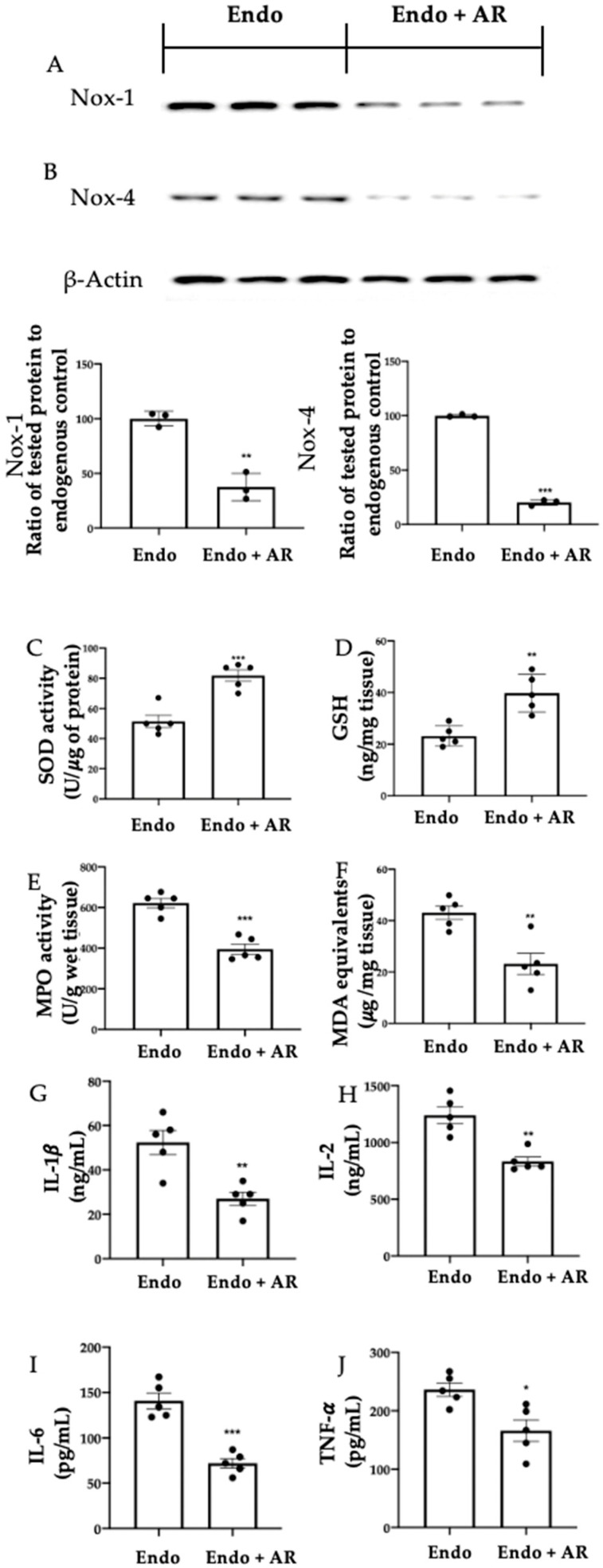
AR administration reduced the oxidative and pro-inflammatory environment. Western blot analysis of Nox-1 (**A**) and Nox-4 (**B**); SOD activity (**C**); GSH levels (**D**); MPO activity (**E**); MDA (**F**), IL-1β (**G**), IL-2 (**H**), IL-6 (**I**), and TNF-α (**J**) levels. The Kolmogorov–Smirnov test was applied to verify the normal distribution of the data, and then the *t*-test was applied. A *p*-value of less than 0.05 was considered significant. * *p* < 0.05 vs. Endo; ** *p* < 0.01 vs. Endo; *** *p* < 0.001 vs. Endo.

Western blot analysis showed reduced Nox-1 (Figure 4A, *p* = 0.0016) and Nox-4 (Figure 4B, *p* < 0.0001) expression in the Endo + AR group (Nox-1 (37.56 ± 7.22), NOX-4 (20.26 ± 1.53)), as compared to the Endo group (Nox-1 (100 ± 3.83), NOX-4 (100 ± 0.71)). Moreover, AR administration restored SOD activity (Figure 4C, Endo group (51.4 ± 4.11), Endo + AR group (81.8 ± 3.73), *p* = 0.0006) and GSH levels (Figure 4D, Endo group (23.2 ± 1.74), Endo + AR group (39.8 ± 3.26), *p* = 0.004), which were impaired in the Endo group. Endo + AR samples also showed reduced MPO activity (Figure 4E, Endo group (622 ± 22.74), Endo + AR group (395.4 ± 24.99), *p* = 0.0002) and lipid peroxidation (Figure 4F, Endo group (43.08 ± 2.58), Endo + AR group (23.18 ± 4.08), *p* = 0.0048), as compared to Endo. AR also reduced inflammation in the lesions, as shown by the reduced IL-1β (Figure 4G, Endo group (52.4 ± 5.42), Endo + AR group (27 ± 2.97), *p* = 0.0034), IL-2 (Figure 4H, Endo group (1240.60 ± 73.16), Endo + AR group (832.8 ± 40.05), *p* = 0.0012), IL-6 (Figure 4I, Endo group (140.8 ± 8.66), Endo + AR group (72 ± 5.32), *p* = 0.0001), and TNF-α (Figure 4J, Endo group (236.2 ± 11.51), Endo + AR group (166.2 ± 18.45), *p* = 0.0123) levels, as compared to the Endo group.

### 2.5. Effect of AR Administration on the Pain Sensitivity Threshold

**Figure 5:**
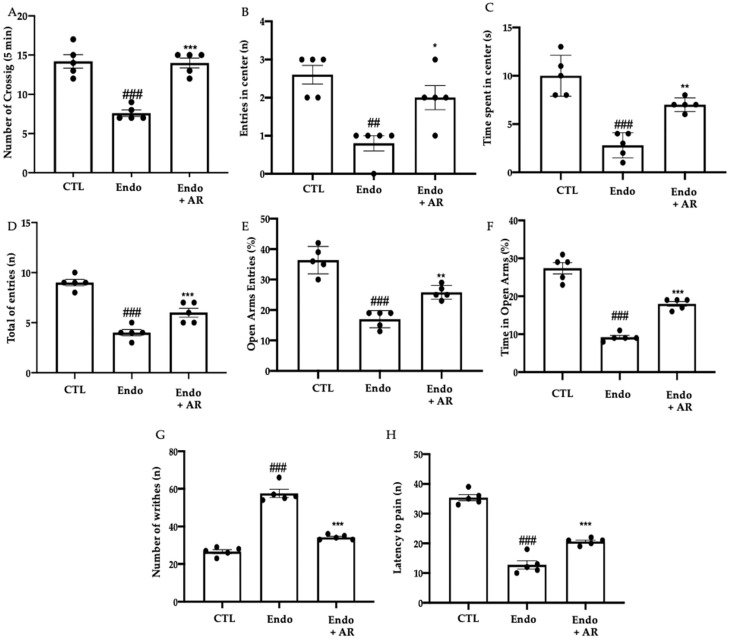
AR administration reduced pain behavior. Open field test: number of crossings (**A**), number of entries in the central square (**B**), and time spent in the central square (**C**); Elevated plus maze test: number of entries in closed and open arms (**D**), % of open entries (**E**), % of time in open arms (**F**), acetic-acid-induced abdominal contractions (**G**), hot plate test (**H**). The results were analyzed by one-way ANOVA followed by a Bonferroni post hoc test for multiple comparisons. A *p*-value of less than 0.05 was considered significant. ## *p* < 0.01 vs. CTL; ### *p* < 0.001 vs. CTL; * *p* < 0.05 vs. Endo; ** *p* < 0.01 vs. Endo; *** *p* < 0.001 vs. Endo.

AR administration restored exploratory behavior and locomotor activity, as compared to the Endo rats (Figure 5A, CTL (14.2 ± 0.86), Endo group (7.6 ± 0.4), Endo + AR group (14 ± 0.63), *p* < 0.0001 vs. CTL, *p* < 0.0001 vs. Endo; Figure 5B, CTL (2.6 ± 0.24), Endo group (0.8 ± 0.45), Endo + AR group (2 ± 0.32), *p* = 0.001 vs. CTL, *p* = 0.0195 vs. Endo; and Figure 5C, CTL (10 ± 0.95), Endo group (2.8 ± 0.58), Endo + AR group (7 ± 0.32), *p* < 0.0001 vs. CTL, *p* = 0.0024 vs. Endo). Additionally, in the elevated plus maze test, the Endo + AR group rats showed a reduced number of entries in closed and open arms (Figure 5D, CTL (9 ± 0.32), Endo group (4 ± 0.32), Endo + AR group (6 ± 0.45), *p* < 0.0001 vs. CTL, *p* = 0.0066 vs. Endo), % of open entries (Figure 5E, CTL (36.4 ± 2.01), Endo group (17 ± 1.26), Endo + AR group (25,8 ± 1.02), *p* < 0.0001 vs. CTL, *p* = 0.0039 vs. Endo) and the % of time in open arms (Figure 5F, CTL (27.4 ± 1.47), Endo group (9.2 ± 0.49), Endo + AR group (18 ± 0.63), *p* < 0.0001 vs. CTL, *p* < 0.0001 vs. Endo), as compared to the Endo group. Endometriosis increased sensitivity to the acetic-acid-induced abdominal contractions (Figure 5G, CTL (26.6 ± 1.03), Endo group (57.6 ± 2.16), Endo + AR group (34.2 ± 0.58), *p* < 0.0001 vs. CTL, *p* < 0.0001 vs. Endo) and to thermal stimuli (Figure 5H, CTL (35.4 ± 1.03), Endo group (12.8 ± 1.39), Endo + AR group (20.6 ± 0.51), *p* < 0.0001 vs. CTL, *p* = 0.0006 vs. Endo), which were significantly reduced by AR administration.

### 2.6. Effect of AR Administration on Neuroinflammation

**Figure 6:**
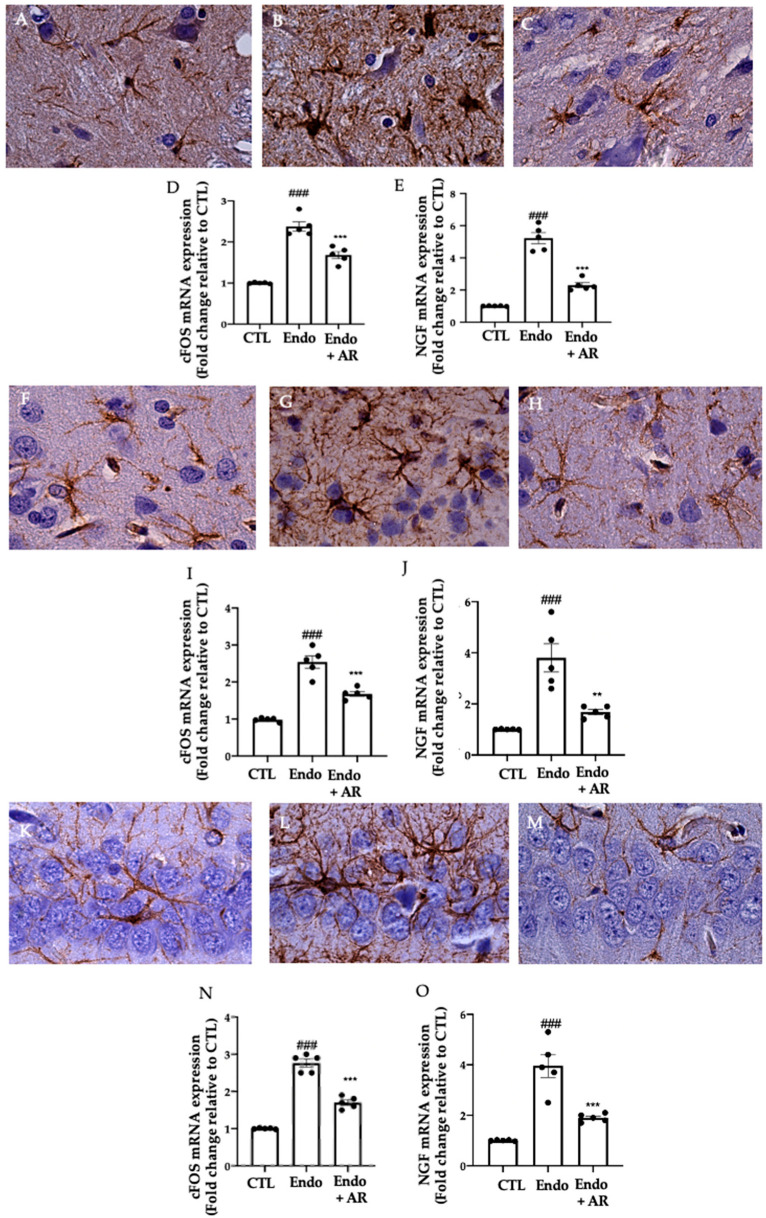
AR administration reduced neurogenic inflammation. Immunohistochemical analysis of GFAP expression in the spinal cord (magnification 100×): Control (**A**), Endo (**B**), Endo + AR (**C**); RT-PCR analysis of: c-FOS (**D**) and NFG (**E**) mRNA levels in the spinal cord; Immunohistochemical analysis of GFAP expression in the brain cortex (magnification 100×): Control (**F**), Endo (**G**), Endo + AR (**H**); RT-PCR analysis of: c-FOS (**I**) and NFG (**J**) mRNA levels in the brain cortex; Immunohistochemical analysis of GFAP expression in the hippocampus (magnification 100×): Control (**K**), Endo (**L**), Endo + AR (**M**); RT-PCR analysis of: c-FOS (**N**) and NFG (**O**) mRNA levels in the hippocampus. The results were analyzed by one-way ANOVA followed by a Bonferroni post hoc test for multiple comparisons. A *p*-value of less than 0.05 was considered significant. ** *p* < 0.01 vs. Endo, ### *p* < 0.001 vs. CTL; *** *p* < 0.001 vs. Endo.

Immunohistochemical analysis was performed to evaluate GFAP expression. Increased GFAP expression was found in the spinal cord (Figure 6B), cortex (Figure 6G), and hippocampus (Figure 6L) of the Endo group, as compared to the control (Figure 6A,F,K). AR administration reduced GFAP immunoreactivity in all tissue examined (Figure 6C,H,M), as compared to the Endo group. RT-PCR showed increased mRNA levels of c-FOS and NGF in the spinal cord (Figure 6D, CTL (1 ± 0.01), Endo group (2.38 ± 0.11), Endo + AR group (1.68 ± 0.09), *p* < 0.0001 vs. CTL, *p* = 0.0001 vs. Endo; and Figure 6E, CTL (1 ± 0.01), Endo group (5.22 ± 0.35), Endo + AR group (2.30 ± 0.16), *p* < 0.0001 vs. CTL, *p* < 0.0001 vs. Endo, respectively), cortex (Figure 6I, CTL (1 ± 0.02), Endo group (2.54 ± 0.17), Endo + AR group (1.68 ± 0.07), *p* < 0.0001 vs. CTL, *p* = 0.0002 vs. Endo; and Figure 6J CTL (1 ± 0.01), Endo group (3.8 ± 0.55), Endo + AR group (1.68 ± 0.1), *p* = 0.0002 vs. CTL, *p* = 0.0016 vs. Endo, respectively) and hippocampus (Figure 6N, CTL (1 ± 0.01), Endo group (2.76 ± 0.11), Endo + AR group (1.7 ± 0.07), *p* < 0.0001 vs. CTL, *p* = 0.0004 vs. Endo; and Figure 6O, CTL (1 ± 0.01), Endo group (3.96 ± 0.46), Endo + AR group (1.9 ± 0.07), *p* < 0.0001 vs. CTL, *p* = 0.0004 vs. Endo, respectively) of the Endo group, as compared to control. Endo + AR group showed reduced c-FOS and NGF mRNA levels in all tissue examined, as compared to the Endo group.

## 3. Discussion

Endometriosis is characterized by a proinflammatory and oxidative environment, hyperproliferation, and dysregulated apoptosis [29]. In this paper, we evaluated the molecular mechanisms of AR administration in endometriosis, focusing on proliferation, oxidative stress, and pain. The disease’s development was monitored by hUS analysis. The administration of AR started once the pathology was established (first hUS analysis). Then, at the end of the experiment, the second hUS analysis showed a reduced lesion diameter, which was confirmed by the macroscopic analysis. AR reduced lesion volume and area. Additionally, there was a significant modification of the lesion histology, with a reduction of glands, stromal tissue, and fibrosis. From a molecular point of view, the smaller size of the lesions corresponds with a reduction in Ki-67 and PCNA expression. These two markers provide important information about cellular cell cycle dysregulation [30,31]. Ki-67 is involved in every phase of the cell cycle except for the G_1_ phase, while PCNA is expressed in the phase of DNA synthesis only, and both are markers of proliferation [32]. AR administration significantly decreased the hyperproliferation that characterized the endometriotic lesions. Many intracellular signaling cascades stimulate cell proliferation. The molecular pathway involved in this stimulation is MAPK. This pathway is significantly disturbed in endometriosis and plays a key role in proliferative signaling. AR confirmed its anti-proliferative effect by reducing ERK and p38 phosphorylation. Hyperproliferation is accompanied by defective control of apoptosis [30]. Our molecular analysis confirmed the impaired expression of the anti- and pro-proteins and DNA fragmentation in the Endo group. AR administration restored the impaired apoptosis by Bax and Bcl-2 expression and the TUNEL assay. AR, apart from its antiproliferative effects, is known for its antioxidant properties. Excessive oxidative stress and depletion of antioxidants are closely associated with endometriosis [33]. Oxidative stress induces hyperproliferation of endometrial stroma, whereas antioxidants may limit stromal proliferation. Several studies have reported a significant decrease in the antioxidant defense, including SOD activity, and an increase in oxidized lipoproteins in the peritoneal microenvironment of women with endometriosis [34]. The increase in SOD activity was a result of oxidative stress, serving as an adaptive cellular response, accompanied by a decrease in GSH levels and an increase in MDA levels. [35]. Rats with endometriosis displayed an activation of the phagocytic cells in the innate immune system, as evidenced by the increased MPO activity in this inflammatory condition [36]. MPO is a critical enzyme of the innate immune system responsible for generating oxidant radicals. The antioxidant properties of AR restored the disturbed balance between oxidants and antioxidants in rats with endometriosis. This was demonstrated by the recovery of GSH levels, the reduction in SOD and MPO activity, and lipid peroxidation. AR administration also resulted in a reduction in the expression of Nox-1 and Nox-4, which are enzymes that play a crucial role in the synthesis of O_2_ and H_2_O_2_. The experimental conditions revealed a close correlation between implant growth, the inflammatory microenvironment, and the manifestation of pain-related symptoms. As previously mentioned, the development of endometriosis is characterized by a significant increase in local inflammation and oxidative stress. This inflammation is observed to increase proportionally with the size of the cyst and the invasion of the peritoneal organs [37]. Recent evidence suggests that endometriosis worsens inflammatory symptoms and affects pain sensitivity [38]. Here, we examined the perception of pain in rats with endometriosis by conducting various tests that assess peripheral and visceral sensitivity. Consistent with previous research, our findings indicate that rats with endometriosis exhibit heightened visceral sensitivity. The animals that underwent endometriosis and were treated with AR showed decreased thermal and mechanical hyperalgesia and pain sensitivity. Endometriosis is associated with both central and peripheral sensitization, leading to increased vulnerability to pain [39]. First, tissue damage and inflammation sensitize the peripheral nociceptive system, causing a decrease in the pain threshold and an increase in the sensory input to the central nervous system. Persistent stimuli can lead to long-term changes in the central nervous system. This phenomenon is known as central sensitization, where the central response becomes disconnected from peripheral input [40]. Chronic pelvic pain and central sensitization can be induced by intensified painful stimuli. The hippocampus is considered one of the key brain regions involved in the emotional and cognitive consequences of neuropathic pain. Abnormal connectivity in the hippocampus and afferences to the frontoinsular and somatosensory cortex were observed in patients with endometriosis [41]. These particular regions of the brain are associated with the shift from short-term, acute pain to long-term, chronic pain [42,43]. AR administration prevented astrogliosis in the spinal cord and hippocampal tissue. Indeed, it strongly reduced the expression of neuroinflammatory mediators.

## 4. Materials and Methods

### 4.1. Animals

Female Sprague–Dawley rats were used in this study. The University of Messina Review Board for Animal Care (OPBA) approved this study. All experiments were performed following the new Italian and EU regulations (D.Lgs 2014/26, EU Directive 2010/63).

### 4.2. Experimental Protocol

The rats were allocated randomly to two groups, one being donors and the other recipients, and endometriosis was induced in accordance with the previously outlined method [44]. To ensure uniform estrogen levels in the rats, the donor animals were given a dose of 10 IU pregnant mare serum gonadotropin. At the 41 h mark, the rats were euthanized, and their uteri were excised. The tissue was finely chopped using scissors and placed in a centrifuge tube of 1.5 mL capacity that contained PBS. The tissue from all the donor rats was combined, and an amount equivalent to one uterus per 500 μL of PBS was administered via intraperitoneal injection along the midventral line of the recipient rats. A period of seven days was allotted for the development of endometriosis. A success rate of 70% was observed for the development of the lesions [45].

### 4.3. Experimental Groups

The rats were allocated randomly and grouped as follows (N = 35 per group):

(1) Endo group: experimental endometriosis was induced in the rats, and they were orally administered with vehicle (saline) using a gavage on the seventh day and subsequently for the following seven days;
(2) Endo + AR group: experimental endometriosis was induced in the rats, and they were orally administered with AR (100 mg/Kg) using a gavage on the seventh day and subsequently for the following seven days;
(3) Control group: the rats were given an intraperitoneal injection of 500 μL of PBS instead of endometrial tissue, and they received a vehicle (saline) via oral gavage on the seventh day and for the subsequent seven days.

**Figure 7:**
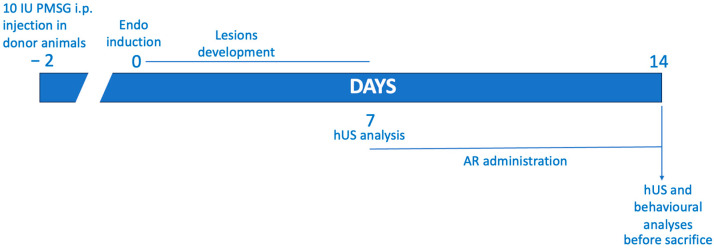
Experimental timeline.

The AR dose was based on previous studies [26]. To assess the impact of administering AR on endometriotic-like lesions, the rats were euthanized 14 days after their induction. Subsequently, a laparotomy was conducted to retrieve the endometriotic implants for additional analyses (Figure 7).

### 4.4. Abdominal High-Frequency Ultrasound

Pelvic ultrasound was performed to monitor the development of the endometriotic lesions at seven and fourteen days after the implant. The analysis included the anterior and posterior pelvic areas to reach the lesions in both locations. Ultrasonographic exams were performed by the Esaote MYLAB OMEGA (Esaote Italia, Milan, Italy) on anesthetized rats (2% isoflurane) positioned in dorsal recumbency. Abdominal B-mode was performed with a high-frequency linear array (4–15 MHz) transducer. Longitudinal and transverse scanning planes were employed for the evaluation of different abdominal structures [46]. All analyses were performed double-blind.

### 4.5. Behavioral Analysis

Behavioral analyses were performed 14 days after the endo induction.

#### 4.5.1. Open Field Test

The measurement of locomotor activity and exploratory behavior was carried out using a square open-field arena [47]. Following a one-minute habituation period, each rat was positioned in one corner of the arena and monitored for five minutes. A 20% ethanol solution was utilized to clean the equipment after each analysis. The recorded parameters included the number of times animals crossed with four legs (spontaneous locomotion), entries into the central square, and time spent in the central square (in seconds).

#### 4.5.2. Hot Plate

The hot plate test was employed to assess the pain threshold to thermal stimuli [48]. The rats were permitted to walk on a hot plate (at a temperature of 53.0 ± 0.1 °C) for a maximum duration of 45 s.

#### 4.5.3. Elevated Plus Maze Test

The apparatus for the elevated plus maze consisted of two enclosed arms and two open arms, which were connected via a central square [49]. The rat was placed in the apparatus and allowed to move around freely for 5 min. A solution containing 20% ethanol was used to clean the apparatus after each analysis. The percentages of total entries, entries in open arms, and time spent in open arms were recorded and reported as % open entries and % time in open arms, respectively.

#### 4.5.4. Acetic-Acid-Induced Abdominal Contractions

The animals were administered an intraperitoneal injection of 0.6% acetic acid, and the number of writhes induced by the acid was observed for 20 min, starting 5 min after the administration [50]. The stretching of the hind limbs followed by a contraction of the abdomen was defined as a writhe.

### 4.6. Abdominal High-Frequency Ultrasound

An ultrasonographic examination was conducted on anesthetized rats (2% isoflurane) placed in dorsal recumbency, using the Esaote MYLAB OMEGA VET. A B-mode ultrasound of the abdomen was carried out using a high-frequency linear array transducer (4–15 MHz) [46]. Both longitudinal and transverse scanning planes were utilized to examine various abdominal structures.

### 4.7. Histological Examination

The endometriotic lesions were fixed in a formaldehyde solution and then embedded in Paraplast [51]. Tissue slides were stained with H&E and evaluated using a Leica DM6 microscope (Leica Microsystems SpA, Milan, Italy). A histological analysis was performed using a double-blind procedure. Histopathological scores were assigned according to the formula P (persistence of epithelial cells in the explants) × I (intensity of glands), as already described [52]. The lesion volume was calculated according to the formula V = (length × width^2^) × 0.5 [53]. Lesions fibrosis was evaluated by Masson trichrome staining (Bio-Optica, Milan, Italy) [54].

### 4.8. Terminal Deoxynucleotidyl Nick-End Labeling (TUNEL) Assay

Apoptosis was analyzed with a TUNEL assay using an in situ cell death detection kit (Roche 11684795910) [55].

### 4.9. Western Blot Analysis

Western blots were performed as already described [56]. The specific primary antibodies anti-PCNA (sc-56, Heidelberg, Germany), anti-p-ERK (sc-7383, Heidelberg, Germany), anti-p-p38 (sc-166182, Heidelberg, Germany), anti-Nox-1 (PA5-103220), and anti-Nox-4 (PA5-72816) were mixed in a 5% *w*/*v* nonfat dried milk solution and incubated at 4 °C overnight. Blots were incubated with a peroxidase-conjugated goat antirabbit IgG (Jackson Immuno Research) or a peroxidase-conjugated bovine antimouse IgG secondary antibody for 1 h at room temperature [57]. To confirm the equal amounts of protein, filters were also incubated with the antibody against β-Actin (sc-47778). Signals were detected with an enhanced chemiluminescence detection system reagent (Super-Signal West Pico Chemiluminescent Substrate) [58]. The relative expression of the protein bands was quantified using densitometry with Bio-Rad ChemiDoc XRS software 2.1.1, #1708265 [59]. Images of the blot signals were imported into analysis software (Image Quant TL, Amersham Biosciences, Freiburg, Germany, v2003) [60].

### 4.10. Biochemical Analysis

The TBARS test was used to assess lipid peroxidation by measuring MDA levels at 535 nm [61]. SOD activity was evaluated as already described [62] and is expressed as U/g protein [63]. GSH levels were determined using a microplate reader at 412 nm [64].

### 4.11. RNA Extraction and cDNA Synthesis

An RNeasy kit (Qiagen, Milan, Italy) was employed to extract RNA for real-time polymerase chain reaction (RT-PCR) analysis. Quantification was performed on RNA with a spectrophotometer (NanoDrop Lite). An iScript RT-PCR kit (Bio-Rad, Hercules, CA, USA) was used to synthesize first-strand cDNA [65].

### 4.12. Real-Time PCR

In total, 1 μL of total cDNA was used to perform RT-PCR analysis with the SYBR Green method (Applied Biosystems). Primer sequences: Bax F:5′-GGTTGCCCTCTTCTACTTT-3′ R:5′-AGCCACCCTGGTCTTG-3′; Bcl-2 F:5′-ACTTTGCAGAGATGTCCAGT-3′ R:5′-CGGTTCAGGTACTCAGCAT-3′; Caspase-8 F:5′-GCGACAGGTTACAGCTCTCC R:5′-ATCAAGCAGGCTCGAGTTGT-3′; Caspase-9 F:5′-CTCAGGCCAGAGGTTCTCAC-3′ R:5′-CAGGAACCGCTCTTCTTGTC-3′; Caspase-3 F:5′-GGCCTGAAATACGAAGTCA-3′ R:5′-GGCAGTAGTCGCCTCTGAAG-3′; cFOS F:5′- 5′-AGCATGGGCTCCCCTGTCA-3′ R:5′-GAGACCAGAGTGGGCTGCA-3′; NGF F: 5′-GACTCCAAGCACTGGAACTC-3′ R: 5′-CACGCAGGCTGTATCTATCC-3′ GAPDH (F:5′-CCATCAACGACCCCTTCATT-3′ R:5′-CACGACATACTCAGCACCAGC-3′) was employed as an internal control [66]. In addition to biological replicates, three technical replicates were carried out for each target gene. To test for potential contamination of genomic DNA in the samples, RNA was used as a template for negative controls in all runs.

### 4.13. Immunohistochemical Analysis

Immunohistochemical localization of anti-GFAP (Proteintech, catalog number 16825-1-AP, dilution 1:5000) was performed in the spinal cord, brain cortex, and hippocampus as already described [67]. All sections were incubated with the primary antibody, then washed with PBS and treated as previously reported [68]. Stained sections were observed using a Leica DM6 microscope (Leica Microsystems SpA, Milan, Italy).

### 4.14. ELISA

IL-1β, IL-2, IL-6 and TNF-α levels were determined using an ELISA kit (BioLegend, San Diego, California; R&D Systems, Milan, Italy) [69].

### 4.15. Statistical Analysis

The Kolmogorov–Smirnov test was applied to verify the normal distribution of the data, and then the *t*-test was applied when comparing the two groups (Prism 8 for macOS version 8.2.1 (279)). In the analyses with three groups, the results were analyzed by one-way ANOVA followed by a Bonferroni post hoc test for multiple comparisons. A *p*-value of less than 0.05 was considered significant. # *p* < 0.05 vs. CTL, ## *p* < 0.01 vs. CTL, ### *p* < 0.001 vs. CTL, * *p* < 0.05 vs. Endo, ** *p* < 0.01 vs. Endo, *** *p* < 0.001 vs. Endo.

## 5. Conclusions

This paper focused on the molecular mechanisms involved in AR administration during endometriosis. AR administration strongly reduced the pathology’s development and the lesion size, showing anti-proliferative and pro-apoptotic effects. The data collected highlighted the AR impact on enhancing the activity of ROS-scavenging enzymes (SOD) and endogenous antioxidant systems (GSH), while suppressing the activity of ROS producing enzymes (Nox). We observed a significant correlation between the inflammatory microenvironment, the growth of endometrial implants, and the development of pain-like symptoms under our experimental conditions. AR administration reduced the expression of neuro-sensitizing mediators, which, in turn, led to reduced activation of astrocytes in the spinal cord, cortex, and hippocampus.

## Data Availability

Based upon the rules of our laboratory, the datasets used in the current study are available from the corresponding author on reasonable request.
